# Psychometric evaluation of a short measure of social capital at work

**DOI:** 10.1186/1471-2458-6-251

**Published:** 2006-10-13

**Authors:** Anne Kouvonen, Mika Kivimäki, Jussi Vahtera, Tuula Oksanen, Marko Elovainio, Tom Cox, Marianna Virtanen, Jaana Pentti, Sara J Cox, Richard G Wilkinson

**Affiliations:** 1 Institute of Work, Health & Organisations, University of Nottingham, 8 William Lee Buildings, Nottingham Science and Technology Park, University Boulevard, Nottingham NG7 2RQ, UK; 2 International Centre for Health and Society, Department of Epidemiology and Public Health, University College London Medical School, 1-19 Torrington Place, London WC1E 6BT, UK; 3 Finnish Institute of Occupational Health, Topeliuksenkatu 42 a A, FIN-00250 Helsinki, Finland; 4 National Research and Development Centre for Welfare and Health (STAKES), POB 220, FIN-00531 Helsinki, Finland; 5 Division of Epidemiology and Public Health, Community Health Sciences, University of Nottingham, QMC, Nottingham NG7 2RD, UK

## Abstract

**Background:**

Prior studies on social capital and health have assessed social capital in residential neighbourhoods and communities, but the question whether the concept should also be applicable in workplaces has been raised. The present study reports on the psychometric properties of an 8-item measure of social capital at work.

**Methods:**

Data were derived from the Finnish Public Sector Study (*N *= 48,592) collected in 2000–2002. Based on face validity, an expert unfamiliar with the data selected 8 questionnaire items from the available items for a scale of social capital. Reliability analysis included tests of internal consistency, item-total correlations, and within-unit (interrater) agreement by *r*_*wg *_index. The associations with theoretically related and unrelated constructs were examined to assess convergent and divergent validity (construct validity). Criterion-related validity was explored with respect to self-rated health using multilevel logistic regression models. The effects of individual level and work unit level social capital were modelled on self-rated health.

**Results:**

The internal consistency of the scale was good (Cronbach's alpha = 0.88). The *r*_*wg *_index was 0.88, which indicates a significant within-unit agreement. The scale was associated with, but not redundant to, conceptually close constructs such as procedural justice, job control, and effort-reward imbalance. Its associations with conceptually more distant concepts, such as trait anxiety and magnitude of change in work, were weaker. In multilevel models, significantly elevated age adjusted odds ratios (ORs) of poor self-rated health (OR = 2.42, 95% confidence interval (CI): 2.24–2.61 for the women and OR = 2.99, 95% CI: 2.56–3.50 for the men) were observed for the employees in the lowest vs. highest quartile of individual level social capital. In addition, low social capital at the work unit level was associated with a higher likelihood of poor self-rated health.

**Conclusion:**

Psychometric techniques show our 8-item measure of social capital to be a valid tool reflecting the construct and displaying the postulated links with other variables.

## Background

In recent years, the concept of social capital has attracted significant attention in public health research. Social capital has been defined in many different ways, but all of them share the notion that networks and norms are important dimensions of the concept. According to one view, social capital refers to those features of social relationships that facilitate collective action for mutual benefit [1,2]. It is therefore seen as a characteristic of social groups rather than individuals, and it is born of shared experience which fosters mutual trust and reciprocity [3]. According to another view, because social capital is created in the connections among individuals in social groups, it is seen as an asset of the individuals [4,5]. However, the health benefits of social capital can be observed both at the individual and collective levels, implemented within a multilevel analytical framework, i.e. individuals nested within areas (or workplaces) that can vary with respect to their levels of social capital [6].

Social capital might be divided into two components. The structural dimension includes social interaction in networks giving access to resources. The values, norms and reciprocity, regarded as the cognitive element of social capital, can be seen as a resource held between individuals interacting within the social networks. In other words, the structural component refers to the extent and intensity of associational links or activity, whereas the cognitive component includes perceptions of support, reciprocity, sharing, and trust [7,8]. However, this distinction may not straightforwardly imply the distinction between individual and structural aspect as it can be argued that individuals might participate in associations and trust can be a characteristic of social structures. Therefore, when it comes to conceptualising and measuring social capital, actions and networks are not necessarily purely structural and cognitive phenomena are not necessarily qualities of individuals. The networks attached to individuals might be resources (capitals) for the achievement of certain outcomes, and the norms of reciprocity, for example, may be embedded in social structure and thus serve as resources for groups.

The two components of social capital – structural and cognitive – have been included in most of the research carried out in the field of social capital and health. However, recently a wide interest in the joint and separate effects of the three types of social capital, namely bonding, bridging and linking social capital, has arisen [9]. While bonding social capital refers to relations between individuals of similar social identity and facilitates cooperation within a group, the term bridging social capital is used to refer to connections between people from different races, classes or ages. Linking social capital, in turn, refers to connections between individuals of different power or status in hierarchies [6]. These constructs are relevant in specifying how social capital inheres in relationships between individuals in similar social context and in different levels of society [7]. Moreover, these three dimensions do not virtually intersect with each other but refer to different ties that cut across different individuals and communities.

The majority of prior studies on social capital and health have assessed social capital in residential neighbourhoods and communities, but the question whether the concept should also be applicable in workplaces has been raised [2,10]. New sources of social capital are likely to be found in settings where people spend most of their time [2]. Therefore, it is evident that workplace is a significant social setting in this sense. Furthermore, it might be hypothesised that all the three elements of social capital could be found at the workplace. Bonding social capital can be seen to refer to workers with similar socio-demographic characteristics, such as similar occupational position or socio-economic status. Bridging social capital, when understood to cut across barriers between people from different races, classes or ages, can be found at workplaces with much diversity. Finally, linking social capital can inhere in the vertical networks between employers and employees with different degrees of institutional power. However, as Putnam [11] has argued, the distinction between these types of social capital, and furthermore the measurement of their separate effects on health should be subjected to further empirical research.

Ziersch [12] has suggested that social capital is multifaceted and its relationship with health is complex. Despite this complexity, the multiple effects of social capital have been extensively studied. However, little research has been conducted to reveal the effects of separate types of social capital on health. Bonding social capital has been associated to better self-rated health [13]. Kim, Subramanian and Kawachi [14] have reported modest effects of community bonding and bridging social capital on health. Moreover, Sundquist and Yang [15] found that low neighbourhood linking social capital was associated with a higher risk of poor health. Low social capital in general has been linked to a range of health outcomes such as higher mortality [16,17], poorer self-rated health [18], and poorer mental health [19]. On the other hand, some other studies have failed to find meaningful relationships between individual-level social capital and self-rated health [20,21].

Strengthening and building social capital has been seen as a potentially important way to reduce socio-economic disparities in health [2,10]. A recent comprehensive review found positive associations between social capital and health with respect to the degree of egalitarianism within a country [22]. Economic equality or inequality of a society might also be seen as a consequence of social capital [11].

The measurement of social capital is subject to considerable debate [3,19,23]. It has been measured by such things as per capita membership in voluntary groups (density of associational membership), interpersonal trust, perceived norms of reciprocity [16], voting behaviours, interaction with neighbours, feelings about helping others [23], as well as by behaviours deemed to be (pro)social such as voter turnout, church membership, and newspaper readership [24]. The heterogeneity of indicators reflects the recency of the concept in health research and the reliance of secondary sources of data [6]. The psychometric properties of these measures have sometimes not been fully evaluated [3,23]. Besides, the existing measures of social capital in residential neighbourhoods and communities might not be adequate and suitable for the work context. As Stone and Hudges [25] have argued, social capital can vary by network type and social scale. Therefore it can be assumed that different measures that tap differentials in settings and cultures might be needed. In addition, shifting the focus of social capital research from residential areas and communities to the contexts where people most interact, such as to the occupational settings and institutions in general, would add important knowledge of the subject. However, research on social capital in work settings is still sparse. This might partly be due to a lack of large data sets with a sufficient number of well-defined workplaces but more importantly due to a lack of psychometrically tested measurable instruments.

It might be hypothesised that within work units, social capital heavily depends on the informal day-to-day and face-to-face interactions between work colleagues, superiors, and subordinates. Furthermore, it would be important to look at not only interaction frequency but also its quality. Similarly as in residential neighbourhoods [2], social capital at work could affect health via processes of informal social control, social cohesion defined as "the extent of connectedness and solidarity among groups in society"[26], maintenance of healthy norms and reinforcing healthy behaviours, and the provision of access to social support. Social capital can be assumed to be gained at the workplace by participating and acting for mutual benefit. Helliwell and Putnam [27] have shown that also greater participation of others increase subjective well-being of the less active.

In sociological and management literature, social capital, defined by such things as structure and context of individuals' networks and density of interaction, has been studied at least in relation to corporate entrepreneurship [28] and mobility [29,30]. Furthermore, one could suggest that the different dimensions of social capital in occupational settings have been measured using a range of various instruments determining concepts such as interactional justice, interpersonal trust, and the quality of team work [31]. All of these instruments, however, include various other features as well and are thus covering conceptually a wide range of phenomena.

The aim of this study is to analyse, in a large sample of Finnish public sector employees, the psychometric properties of a measure based partly on existing instruments that could assess the core dimensions of social capital.

## Methods

This study is based on cross-sectional data obtained from the Finnish Public Sector Study, an ongoing prospective study to explore the relation of behavioural and psychosocial factors with health [32,33]. The study is focused on the entire personnel of ten towns and 21 hospitals in the areas where the towns are located (*N *= 70,961). Similar methods of data collection were used in both sub-samples, in the 10-Town Study [32] and in the Hospital Personnel Study [33]. All workers employed in these organisations were invited to participate and participation was voluntary. In 2000–2002, 32,293 municipal and 16,299 hospital employees (81% women) aged 17 to 65 responded to a postal questionnaire survey with a response rate of 68%. The sample covers almost 20% of the full-time employees working in Finnish municipal sector. The participants represented more than 1800 occupational titles and. The most common occupations of the respondents were registered nurse (23%, *N *= 10,990), teacher (19%, *N *= 9315), practical nurse (13%, *N *= 6221) and cleaner (10%, *N *= 4659).

The sample did not substantially differ from the eligible population. In the ten town sub sample, figures for participants vs. eligible population (*N *= 47,351) were as follows: mean age 44.9 vs. 44.5 years, proportion of women 77% vs. 72%, proportions of upper non-manual, lower non-manual and manual employees 34%, 46%, 20% vs. 35%, 42% and 22%, respectively. The corresponding figures for the hospital sub sample (*N *= 23,610) were: mean age 43.1 vs. 43.1 years, proportion of women 87% vs. 84%, proportions of upper non-manual, lower non-manual and manual employees 16%, 77%, 8% vs. 13%, 81% and 7%, respectively.

In the Finnish Public Sector Study, written consent was obtained from the participants for linking register-based information on sickness absences with survey responses. Regarding the questionnaire survey (the present data), written consent was not obtained as the study was approved by the Ethics Committee of the Finnish Institute of Occupational Health.

### Measures

A questionnaire survey was used as the source of test items by which social capital at work was assessed and other constructs derived to be used as validity criteria.

#### Social capital at work

The items of social capital at work were selected on the basis of an inequality perspective of the efficacy of social capital [10]. These items indicate whether people feel that they are respected, valued and treated as equals at work, rather than feeling that it is all a matter of seniority in their hierarchy. The scale was designed to assess social capital specifically in work context. The selected eight Likert-scaled items (range of scales 1–5) are presented in Table 1.

The scale measures both the cognitive and structural components of social capital. Cognitive social capital, which refers to beliefs, attitudes and values such as trust, solidarity and reciprocity that are shared among members of the same community, was measured with items 3,5 and 8. Structural social capital, which is formed through horizontal organizations and networks that have collective and transparent decision making processes, accountable leaders, practices of collective action and mutual responsibility, was measured with items 1, 2, 4, 6 and 7. The scale also taps, to some extent, the three forms of social capital. Items 3, 4 and 5 measure predominantly bonding social capital, whereas items 6 and 7 comprise co-operative relations and mutuality needed for "getting ahead" indicating the core perception of bridging social capital [34]. Items 1, 2 and 8 assess the trusting relationships between people of different authority gradients and thereby measure the linking social capital at work.

A summary score of ratings of all social capital items was constructed. A high score in the scale indicates high social capital.

The dataset included individuals (employees) nested within work units in towns and hospitals. Therefore, we constructed aggregated social capital scores according to the work units in addition to individual scores. The work unit of each respondent was identified from the employers' records based on a five-level organisational hierarchy classification. Work unit was the lowest level in the organisational hierarchy. Work unit level social capital was calculated as the mean of individual level responses. This aggregated variable, social capital at work, was then assigned to the respective unit and linked to each member of the work unit. If the number of respondents in the work unit was less than three, these respondents were excluded from the analysis (*N *= 2225). Thus, in all cases, the work unit level scores for social capital were based on values derived from three or more individual respondents.

#### Procedural organisational justice

Procedural justice refers to the extent to which employees are treated with justice at their workplace and indicates whether decision-making procedures include input from affected parties, are consistently applied, suppress bias, and are accurate, correctable, and ethical. The evaluation scale was adopted from Moorman [31] (7 items, Cronbach's alpha 0.90). The scale measures the extent to which managerial procedures promote consistency, bias suppression, accuracy, correctability, representativeness, and ethicality. (Example items: 'Procedures are designed to collect accurate information necessary for making decisions', 'Procedures are designed to provide opportunities to appeal or challenge the decision'.) The response options were from 1 = totally disagree, to 5 = totally agree.

#### Trait anxiety

Trait Anxiety Inventory [35] (6 items; Cronbach's alpha = 0.88) was used to measure negative affectivity, which is the disposition to answer negatively to questionnaires. (Example items: 'I feel nervous and restless', 'I feel inadequate'.) The response options were from 1 = almost never, to 4 = almost always.

#### Job control

Job control concerns decision authority and skill discretion in work. The measure was derived from the Job Content Questionnaire [36,37]. Job control was assessed with nine questions about the worker's ability to use and develop skills and exert decision authority (Cronbach's alpha = 0.82). (Example items: My job requires that I learn new things', My job allows me to make a lot of decisions on my own'.) The responses were given on a Likert scale of 1 = "very little" to 5 = "very much". The total score for the construct was computed.

#### Effort-reward imbalance

According to the effort-reward imbalance model, health risk derives from the mismatch between efforts expended at work and rewards received in turn in terms of money, social approval, job security, and career opportunities. A standard measure of effort-reward imbalance (ERI) [38] in Finnish was not available in this study. The questionnaire used included one question about effort in work and three questions about rewards. These measures were used to construct the proxy measure of ERI. Effort in work was measured with the following question: "How much do you feel you invest in your job in terms of skill and energy?" Rewards were assessed with a scale containing three questions about feelings of getting in return from work in terms of (1) income and job benefits, (2) recognition and prestige, and (3) personal satisfaction (Cronbach's alpha = 0.64) [39,40]. Response format for all the questions was a five-point Likert scale ranging from 1 = "very little" to 5 = "very much". The indicator of effort-reward imbalance was obtained by calculating the ratio between the response score in the effort scale and the mean response score in the reward scale.

If half or more of the component items were missing, a value of missing was recorded in the total scores of social capital, procedural justice, job control, and rewards.

#### Magnitude of change in work

This was measured with a single question assessing the changes in one's work ('When you think about all the changes which have happened in your work during the past year, how would you describe the situation from your point of view?'). The respondents evaluated the magnitude of changes with a 7-point scale ranging from small and insignificant to large and significant.

#### Self-rated health

Poor self-rated health was indicated by health ratings less than good on a 5-point single item scale: "How would you estimate your current state of health?" [41]. The measure was dichotomised by grouping the response scores 1–3 into the category of poor self-rated health and scores 4–5 into the category of good self-rated health. A total of 26% the participants were classified as having poor self-rated health.

### Statistical analysis

A range of psychometric methods were used to evaluate the reliability and validity of the measure of social capital at work.

First, the internal consistency (Cronbach's alpha) of the scale was calculated. An alpha-value greater than 0.7 indicates a satisfactory internal consistence for a scale [42].

Second, an aspect of convergent validity was explored by analysing the association of social capital at work with other work-related constructs such as procedural justice, effort-reward imbalance, and job control. Similarly to social capital, the concepts of procedural justice, effort-reward imbalance and job control are contextual characteristics of psychosocial work environment that are assumed to influence the health of employees, and can thus be seen as theoretically related. Indeed, numerous studies have shown that these factors are associated with health outcomes, such as self-rated health.

Third, divergence validity was assessed in relation to trait anxiety and magnitude of changes in work. Trait anxiety is a characteristic of a person rather than of the work environment. Change in work, in turn, may be determined by a large range of different physical and psychosocial factors, not only by those specific psychosocial factors that are expected to influence health. Multilevel regression modelling was used.

Fourth, to test within-unit (interrater) agreement, that is, the extent to which raters assign the same ratings to a single target, we computed a *r*_*wg *_index, which measures agreement on a single-item scale [43,44]. This index compares the observed variance in the raters' responses to the variance that would be expected if the ratings were characterized by uniformly distributed error. A *r*_*wg *_equal to 1 would indicate that all judgements about rated subject were similar. The more there is decline in the *r*_*wg *_index close to 0, the wider there is the divergence of the opinions on the issue. When the index is 0, the suitability to use aggregated individual-level scores as indicators of group-level constructs is less obvious or unsubstantial. Therefore, a demonstration of interrater agreement further provides the measurement justification for using aggregated individual-level data as indicators of group-level constructs. An *r*_*wg *_index value of 0.7 is perceived as a limit value.

Fifth, the construct validity of aggregated lower-level measures as representations of higher-level constructs is generally addressed through examining patterns of within-group variance, for example intra class correlation (ICC). The ICC evaluates between-group variance relative to total (between and within) variance [45]. As variance can only be positive, the ICC receives values between 0 and 1. An increase in ICC indicates the addition of individual differences that are at the work unit level. An ICC of 0 would suggest that the work units are similar to random samples and are not relevant to understanding differences in social capital.

Finally, criterion-related validity of the social capital measure was assessed with the measure of self-rated health [38]. Sex-specific age-adjusted odds ratios (ORs) and their 95% confidence intervals (CIs) for poor self-rated health were obtained from logistic regression models for both the individual-level and work unit level (aggregated) scores of social capital at work.

The multilevel analyses were performed by using the GLIMMIX procedure in SAS 9.1.3 (SAS Institute, Inc., Cary, North Carolina) software package. The GLIMMIX procedure fits statistical models to hierarchical data with correlations or nonconstant variability. It allows for simultaneous examination of the effects of the individual and group level variables on individual level outcome, while accounting for the non-independence of observations within groups.

## Results

The mean and standard deviation for each of the 8 social capital items is presented in Table 1 separately for the women and the men. Mean values of the social capital items were slightly higher for the women than for the men.

### Reliability

The item-item and item-total correlations as well as Cronbach's alpha values of the scale if item is deleted are given in Table 2. The internal consistency of the scale was good (Cronbach's alpha = 0.88 for the women and 0.88 for the men). The item-total correlations were in the range of .58 to .69 (*p *< 0.001). Correlations between items varied between .28 and .80 (*p *< 0.001). The presence or removal of any of the items did not materially alter the internal reliability of the scale.

To test within-unit agreement, we computed *r*_*wg *_index for the social capital measure. In our sample, the average deviation of an individual's perception of social capital from the mean level of her/his work unit was only 0.88, indicating a significant homogeneity in the perceptions of social capital within a work unit and supported the aggregation of unit members' social capital to the work unit level.

### Validity

The face validity (intuitive appeal) of our measure appears credible as it encompasses both structural and cognitive components of social capital and does not measure any outcomes of social capital.

We tested construct validity by assessing intra class correlation (ICC) as a measure of resemblance between individuals belonging to the same work unit. A high ICC value indicates that work units are very important in understanding the contextual effects of social capital. However, a high ICC might also be attributable to different composition of work units. In our sample ICC = 21%, which strongly suggests that work units are important determinants of social capital.

An aspect of construct validity was tested by exploring the associations of social capital with theoretically related constructs of procedural justice, effort-reward imbalance, and job control using multilevel linear regression. The divergence validity was assessed in relation to theoretically unrelated concepts of trait anxiety and magnitude of changes in work. Table 3 shows that as expected, the measure of social capital was significantly positively associated with theoretically related constructs of procedural justice (β = 0.53 for the women and β = 0.65 for the men) and job control (β = 0.28 for the women and β = 0.29 for the men) and negatively associated with effort-reward imbalance β = -0.23 for the women and β = -0.25 for the men) (*p *< 0.001 in all cases). In contrast, the associations with trait anxiety and magnitude of changes in work were much weaker.

We tested the criterion-related validity in relation to self-rated poor health. As Table 4 shows, in multilevel logistic regression models, significantly elevated odds ratios (ORs) of poor self-rated health were obtained for the participants in the lowest quartile of social capital (OR = 2.42, 95% confidence interval (CI): 2.24–2.61 for the women and OR = 2.99, 95% CI: 2.56–3.50 for the men). Further adjustment for personality factor trait anxiety had no effect on the associations. Adjustment for SES slightly attenuated the association in men (OR = 2.77, 95% CI: 2.36–3.24). (Data not shown.)

As displayed in Table 5, lower work unit level (aggregated) social capital was also associated with poor self-rated health in multilevel logistic regression models, although the association was weaker than at the individual level. Among the women, the OR in the lowest quartile of work unit social capital was 1.19 (95% CI: 1.10–1.30). The corresponding figure for the men was 1.79 (95% CI: 1.51–2.11). Further adjustment for SES attenuated the association in men (OR = 1.48, 95% CI: 1.26–1.75). (Data not shown.)

## Discussion

There has been a need for valid and reliable research tools to examine social capital at work in relation to health outcomes. In general, no consensus has yet been reached how to measure social capital [3] and few of the indicators or scales used to measure social capital have been subjected to widespread and standard psychometric testing [23]. Moreover, different settings such as work settings and residential areas need different measures.

The results of this investigation in a large sample of Finnish public sector employees reveal good psychometric properties of an 8-item scale measuring social capital at work. Internal consistency, item-item correlations, item-total correlations, and reliability if item deleted were evaluated. These justified that the items form a scale.

In our measure of social capital, three items (1, 2 and 8) assessed vertical or institutional trust in the supervisor, while the remaining four items (3–7) mainly measured horizontal trust in workmates, although items 4 and 6 to some extent also indicated membership in local horizontal networks (the participation/network aspect of social capital). Therefore, the score on the constructed total social capital variable was dependent on the proportion of items reflecting vertical trust in the supervisor as opposed to the proportion reflecting horizontal trust in workmates and horizontal networks. This raises the question whether all 8 items should be combined into one index variable. However, since the item-total correlations were sufficiently high and our further analysis showed that the presence or removal of any of the items did not materially alter the internal reliability of the total scale, we argue that it is justified to use the total measure.

Previous research has assessed social capital at work in relation to health using non-validated measures. In their study on social capital and mortality in Russia, Kennedy, Kawachi and Brainerd [46] used quality of work relations (conflicts in workplace) as an indicator of social capital. Veenstra [20] performed an analysis of the relationship between individual level social capital and self-rated health using social engagement (frequency of socialisation with co-workers and willingness to turn to a co-worker in times of trouble) as a proxy for social capital at work. Liukkonen et al. [47] measured social capital by security of employment contract and trust in co-worker support, whereas Lindström et al. [48] measured participation in activities such as study circle at work or union meetings.

We assessed criterion-related validity of the 8-item social capital measure by checking it against of a relevant outcome [49], poor self-rated health [38], which has been associated with lower social capital in prior studies [18]. Significantly elevated odds ratios of poor health were observed for the employees in the lowest quartile of social capital. The significant association in multilevel models together with the substantially weak association with one personality measure (trait anxiety) can indicate that the construct of social capital might be more than an aspect of an individual's personality. In other words, the measure seems to tap characteristics that vary at a work unit level and are not solely explained by individual differences.

As the test of criterion validity was based on cross-sectional design, observed associations could reflect predictive validity, but also reverse causation and common method variance. Low levels of social capital might lead to poorer health, but poor health could also weaken a worker's social capital and response set could artificially inflate associations between social capital at work and self-rated health in such data. Self-report measures can be subject to reporting bias and may be influenced by specific personality characteristics and response styles. In consequence, it is possible that poor health leads to lower social capital, or that self-rated health status and social capital are both expressions of people's general well-being [13].

However, both reversed causality and common method variance are less likely explanation for results based on work unit level social capital indicator, as the level of a person's health is unlikely to influence such scores. Furthermore, the significant association between individual-level social capital and poor self-rated health did not change after adjustment for a personality factor trait anxiety.

We were unable to assess concurrent validity (the ability to distinguish between groups that should be different) as previous studies have not found consistent differences in levels of social capital between women and men, SES groups, or other groups [19]. Moreover, convergent validity (agreement with results from other instruments) is not currently possible to fully assess as no established 'gold standard' measure of social capital at work is available. However, the aspect of construct validity was assessed examining the associations of social capital with theoretically related and unrelated constructs. The scale was associated with, but was not redundant to, conceptually close constructs, such as procedural justice, job control, and effort-reward imbalance and its associations with conceptually more distant concepts were weaker, supporting the construct validity of the scale [49].

### Strengths and limitations

The face validity of our measure appears credible. In contrast to many indicators of work-related social capital used in previous studies, the measure taps several key aspects of the concept including membership in local networks, trust, collective action, diversity and tolerance [50], sense of belonging and sense of fairness. However, our measure did not directly tap the negative aspects or the indicators of lack of social capital, such as bullying, hostility, or poor organisational climate, as these aspects were not covered by the data of the Finnish Public Sector Study. In future studies, complementary indicators with the 8-item scale should be needed if direct assessment of these negative aspects is seen as crucial.

Finally, even if our sample was large and heterogeneous, it remains unclear to what extent the reported psychometric properties hold true in other countries and for private sector samples. Therefore, more research in other countries and with other samples is needed to further validate this measure.

## Conclusion

Although accurate measurement is a key requisite for scientific progress, there has been little standardisation in the assessment of social capital complicating interpretation of inconsistent evidence and increasing the possibility of selective publication. For the work context, no standard validated measure of social capital has been available. The present findings support the notion that social capital at work is a meaningful construct and is associated with poor self-rated health. Moreover, psychometric techniques to explore reliability and validity show that our 8-item measure is a valid tool reflecting the construct and displaying the postulated links with other variables.

## Abbreviations

AD = average deviation; CI = confidence interval; ERI = effort-reward imbalance; ICC = intra class correlation; OR = odds ratio; SD = standard deviation.

## Competing interests

The author(s) declare that they have no competing interests.

## Authors' contributions

The Finnish Public Sector study is a large multicentre study with several collaborators. AK designed the study, carried out the data analyses and was the principal author of the paper. MK and JV are the directors of the Finnish Public Sector Study and the second principal investigators of the study; they helped in designing the study, the data analysis, interpreting the results, and writing the paper. TO, ME, TC and SJC contributed to interpretation of the results and manuscript writing. MV collected the data, contributed to interpretation of the results, and edited the manuscript. JP constructed the measures, helped in designing the study and supervised the data analyses. RGW helped in designing the study, selected the social capital items, contributed to interpretation of the results and writing the paper.

All authors read and approved the final manuscript.

## Pre-publication history

The pre-publication history for this paper can be accessed here:



## Figures and Tables

**Table 1:** Social capital item means and standard deviations

	*Women *(*N *= 38,914)	*Men *(*N *= 9337)
Item 1. Our supervisor treats us with kindness and consideration.	3.85 (1.08)	3.77 (1.08)
Item 2. Our supervisor shows concern for our rights as an employee.	3.82 (1.06)	3.78 (1.08)
Item 3. We have a 'we are together' attitude.	3.60 (1.05)	3.41 (1.09)
Item 4. People keep each other informed about work-related issues in the work unit.	3.85 (0.92)	3.68 (0.99)
Item 5. People feel understood and accepted by each other.	3.29 (1.03)	3.20 (1.02)
Item 6. Do members of the work unit build on each other's ideas in order to achieve the best possible outcome?	3.38 (0.89)	3.23 (0.92)
Item 7. People in the work unit cooperate in order to help develop and apply new ideas.	3.25 (1.05)	3.08 (1.06)
Item 8. We can trust our supervisor.	3.76 (1.17)	3.78 (1.15)

*Note*: 1 = fully disagree; indicative of low social capital, 5 = fully agree; indicative of high social capital; except item 7 where 1 = very little 5 = very much.

**Table 2:** Item-item correlations*, item-total correlations* and Cronbach's alphas if item deleted: social capital items

	*Item 1*.	*Item 2*.	*Item 3*.	*Item 4*.	*Item 5*.	*Item 6*.	*Item 7*.	*Item-total correlation*	*Alpha if item deleted*
*Women *(*N *= 36,779)									
Item 1.								.61	.86
Item 2.	.78							.66	.86
Item 3.	.32	.35						.69	.85
Item 4.	.28	.31	.66					.58	.86
Item 5.	.36	.38	.68	.54				.67	.86
Item 6.	.30	.34	.56	.46	.53			.60	.86
Item 7.	.32	.35	.63	.50	.57	.60		.64	.86
Item 8.	.72	.78	.36	.31	.39	.34	.35	.64	.86
									
*Men *(*N *= 8792)									
Item 1.								.63	.87
Item 2.	.80							.67	.87
Item 3.	.34	.37						.69	.86
Item 4.	.32	.34	.66					.62	.87
Item 5.	.38	.40	.67	.56				.67	.86
Item 6.	.33	.37	.57	.49	.54			.62	.87
Item 7.	.33	.36	.64	.53	.57	.62		.65	.87
**Item 8**.	.73	.79	.38	.35	.40	.36	.37	.65	.87

Note: *p *< 0.001 in all cases.

*Pearson correlations.

**Table 3:** Associations between social capital measure and other constructs (GLIMMIX)

	Women		Men	
	*N*	β	*N*	β
Procedural justice	35,976	0.53	8642	0.65
Effort-reward imbalance	30,560	-0.23	7756	-0.25
Job control	36,986	0.28	8761	0.29
Trait anxiety	36,397	-0.07	8612	-0.14
Magnitude of change in work	36,052	-0.02	8631	0.07

Note: *p *< 0.001 in all cases, except magnitude of change in work where p = 0.071 in women and p = 0.002 in men.

**Table 4:** The relationship between individual level social capital and poor self-rated health.

	*N*	*Odds ratio (95% CI)*
*Women*:	36,771	
*Social capital (quartiles)*		
1 (highest)		1.00
2		1.36 (1.27–1.47)
3		1.67 (1.55–1.79)
4 (lowest)		2.42 (2.24–2.61)
*Men*:		
*Social capital (quartiles)*	8709	
1 (highest)		1.00
2		1.32 (1.12–1.54)
3		1.69 (1.45–1.98)
4 (lowest)		2.99 (2.56–3.50)

Age adjusted odds ratios and their 95 percent confidence intervals (95% CIs) from multilevel logistic regression models.

**Table 5:** The relationship between work unit level social capital and poor self-rated health.

	*N*	*Odds ratio (95% CI)*
*Women*:		
*Social capital (quartiles)*	37041	
1 (highest)		1.00
2		1.13 (1.04–1.23)
3		1.25 (1.15–1.36)
4 (lowest)		1.19 (1.10–1.30)
*Men*:		
*Social capital (quartiles)*	8777	
1 (highest)		1.00
2		1.22 (1.01–1.47)
3		1.31 (1.09–1.57)
4 (lowest)		1.79 (1.51–2.11)

Age adjusted odds ratios and their 95 percent confidence intervals (95% CIs) from multilevel logistic regression models
