# Correction: psychometric evaluation of a short measure of social capital at work

**DOI:** 10.1186/1471-2458-7-90

**Published:** 2007-05-23

**Authors:** Anne Kouvonen, Mika Kivimäki, Jussi Vahtera, Tuula Oksanen, Marko Elovainio, Tom Cox, Marianna Virtanen, Jaana Pentti, Sara J Cox, Richard G Wilkinson

**Affiliations:** 1 Institute of Work, Health & Organisations, University of Nottingham, 8 William Lee Buildings, Nottingham Science and Technology Park, University Boulevard, Nottingham NG7 2RQ, UK; 2 Department of Epidemiology and Public Health, University College London Medical School, 1-19 Torrington Place, London WC1E 6BT, UK; 3 Finnish Institute of Occupational Health, Topeliuksenkatu 42 a A, FIN-00250 Helsinki, Finland; 4 National Research and Development Centre for Welfare and Health (STAKES), POB 220, FIN-00531 Helsinki, Finland; 5 Division of Epidemiology and Public Health, Community Health Sciences, University of Nottingham, QMC, Nottingham NG7 2RD, UK

In review of our article (Kouvonen et al: *BMC Public Health *2006, 6:251) we found an error in the equation of our measurement of trait anxiety.

Our data are comprised of two sub-samples, the 10-Town Study and the Hospital Personnel Study. Similar methods of data collection were used in both sub-samples. However, the direction of response alternatives for one of the scales (trait anxiety) differed between the two sub-samples.

That is to say that the response alternative "almost never" was coded as "1" in the first sub-sample whereas in the second sub-sample it was coded as "4". Therefore we can also deduce that the responses of the participants from the different sub-samples were coded into the different direction.

This error is corrected by recoding the variable so that in both sub-samples the higher score indicates higher trait anxiety. We re-conducted the analyses and found only minor differences compared to the original figures given and we also found that the error did not alter the main results or conclusions drawn.

The following corrections should be incorporated into any future analysis of our original article [1].

Within the Methods section, paragraph 10 (*Trait anxiety*), the Cronbach's Alpha for Trait Anxiety should be 0.85 instead of 0.88.

Within the Results, paragraph 6 (2^nd ^paragraph within *Validity*), the last sentence should now read as "The associations with trait anxiety and magnitude of changes in work were weaker." rather than "In contrast, the associations with trait anxiety and magnitude of changes in work were much weaker."

Within the Results, paragraph 7 (3^rd ^paragraph within *Validity*), the 3^rd ^sentence should read "Further adjustment for personality trait anxiety attenuated the association in both sexes, but the results remained statistically significant." rather than "Further adjustment for personality factor trait anxiety had no effect on the associations."

Within the original Table 3 (The corrected version is available with this article as Table 1), the following two figures should be revised; Trait anxiety women should be changed from β = -0.07 to β = -0.19; and for men should be changed from β = -0.14 to β = -0.22.

**Table 1:** Corrected table 3: Associations between social capital measure and other constructs (GLIMMIX)

	Women		Men	
	*N*	β	*N*	β

Procedural justice	35,976	0.53	8642	0.65
Effort-reward imbalance	30,560	-0.23	7756	-0.25
Job control	36,986	0.28	8761	0.29
Trait anxiety	36,397	-0.19	8612	-0.22
Magnitude of change in work	36,052	-0.02	8631	0.07

Note: *p *< 0.001 in all cases, except magnitude of change in work where p = 0.071 in women and p = 0.002 in men.

And finally, within the discussion, paragraph 5 (paragraph beginning "We assessed criterion") The 3^rd ^sentence should read "The significant association in multilevel models together with a weaker association with one personality measure (trait anxiety) can indicate that the construct of social capital might be more than an aspect of an individual's personality." rather than "The significant association in multilevel models together with the substantially weak association with one personality measure (trait anxiety) can indicate that the construct of social capital might be more than an aspect of an individual's personality."

## Pre-publication history

The pre-publication history for this paper can be accessed here:


